# Characterization of the Aromatic and Phenolic Profile of Five Different Wood Chips Used for Ageing Spirits and Wines

**DOI:** 10.3390/foods9111613

**Published:** 2020-11-06

**Authors:** María Guerrero-Chanivet, Manuel J. Valcárcel-Muñoz, M. Valme García-Moreno, Dominico A. Guillén-Sánchez

**Affiliations:** 1 Departamento de Química Analítica, Facultad de Ciencias, Instituto Investigación Vitivinícola y Agroalimentaria (IVAGRO), Campus Universitario de Puerto Real, Universidad de Cádiz, 11510 Cádiz, Spain; maria.guerreroch@uca.es (M.G.-C.); dominico.guillen@uca.es (D.A.G.-S.); 2 Bodegas Fundador S.L.U., Departamento de Investigación y Desarrollo, C/San Ildefonso, n° 3, 11403 Cádiz, Spain; m.valcarcel@bodegasfundador.com

**Keywords:** oak, cherry, chestnut, wood chips, phenolic compounds, aroma, ageing

## Abstract

Wooden barrels and wood chips are usually used in the ageing of spirits and wines to improve their sensorial profile. Oak wood is the most popular material used in cooperage, but there are other interesting woods, such as cherry or chestnut, that could be considered for this purpose. In this study, a novel method for the determination of the aromatic profile of wood powder by Direct Thermal Desorption-Gas Chromatography-Mass Spectrometry (DTD-GC-MS) was optimized by experimental design. The volatile composition of five different types of wood chips was determined by direct analysis of wood powder by DTD-GC-MS method developed. Thirty-one compounds from wood were identified through this analysis, allowing the differentiation between woods. The aromatic and phenolic compound profile of the 50% hydroalcoholic extract of each type of wood studied was analyzed by Stir-bar Sorptive Extraction-Gas Chromatography-Mass Spectrometry (SBSE-GC-MS) and Ultra-High-Performance Liquid Chromatography (UHPLC) to determine which wood compounds are transferred to spirits and wine after ageing. Different phenolic profiles were found by UHPLC in each wood extract, allowing their differentiation. However, results obtained by SBSE-GC-MS did not allow distinguishing between wood extracts. The analysis of wood in solid state, without any type of previous treatment except grinding, by DTD-GC-MS does not imply any loss of information of the aromatic compounds present in wood as other techniques. This is a potential method to identify aromas in wood that, in addition, allows different types of wood to be differentiated.

## 1. Introduction

Ageing spirits and wines in wooden barrels or the use of wood chips are industrial common practices that change the sensorial profile of the product. The structural characteristics and chemical composition of the wood are responsible for many of the processes that take place during the maturation period, affecting the composition of the spirits and wines, modulating their sensorial quality and complexity, such as aroma, structure or astringency and contributing to their stability. Wood characteristics, such as the geographical origin and botanical species [1,2,3,4,5,6], volume of the barrel [7] or chip size [8,9] and toasting level [3,10,11], affected the sensorial profile of the final product. There are many spirits such as armagnac, cognac, brandy, whisky, rum, tequila or grappa as well as wines, that are aged in barrels or use wood chips in their ageing processes in order to obtain a special aroma profile.

The barrel or the chips are key elements during the ageing process. They are active contributors to the sensorial properties of the distillates that are in touch with them. There are several physical-chemical phenomena where components of the spirit (made from wine, cane sugar, malt, agave, etc.) or the wine compounds from the wood are involved [10,12,13]. Most of them are extraction processes, but other chemical reactions take place, such as oxidation, esterification, hydrolysis, ethanolysis, Maillard reactions, polymerization, and polycondensation reactions. There are also physical phenomena, as evaporation or the perspiration of water molecules to the outside through the wood, that take place too during ageing process. All of them depend on many variables, as the composition of the wood, the atmospheric conditions or the type of distillate and its alcoholic strength.

Wood is composed, mostly, of holocelluloses (cellulose and hemicellulose) and lignin. They represent around 90% of the total of wood. There are other compounds, as phenolic compounds (polyphenols or simple phenols), fatty acids, alcohols or inorganic substances, that represent 10% of the wood composition [13]. Wood can play a significant role in contributing flavor to alcoholic beverages. Most of these compounds are responsible for the sensorial profile of the final product. Lignin is a polymer that can suffer thermal degradation during the manufacturing of barrels or by ethanolysis and hydrolysis during spirit and wine maturation powered by their acid character [14]. Compounds from hemicellulose as furfural and derivatives [15,16] and compounds from lignin as guaiacyl-type aldehydes (vanillin and coniferylaldehyde), syringyl-type aldehydes (syringaldehyde and sinapaldehyde), and cinnamic and benzoic acids [10,13] are the most significant components extracted from wood during maturation. Other compounds, as hydrolysable tannins, as gallotannins and ellagitannins, are highly soluble in ethanol-water solutions and their transformation into gallic acid or ellagic acid by hydrolysis is very common [13].

The geographical origin and botanical species affect the composition of the wood. Oak is the main material used in cooperage to make barrels as well as wood chips destined to aged spirits and wines, but also chestnut and cherry are used for this purpose. Traditionally, American and French oak wood (*Quercus alba* and *Quercus petraea* and *robur*) are the most employed type of wood in cooperage companies to make barrels and wood chips. Chestnut wood is characterized by a higher porosity than oak, and high quantities of polyphenols may be transferred to the distillate. Cherry wood is characterized by a high porosity too and oxygen permeation, and is usually used for short ageing times [17]. Chestnut wood has proved to be as sustainable for cooperage and has interesting properties for the ageing of brandies [18,19] and could be interesting to age other spirits and wines. As regards cherry wood, it has also been considered as a possible source of wood for the production of wines or spirits [20,21,22,23].

References related to the direct analysis of wood in order to characterize its chemical and aromatic profile were not found in the bibliography. It is interesting to know which compounds are present in wood and if they could be transfer to the alcoholic beverage during its ageing. The main goal of this work is to know the aromatic and phenolic profile of five different wood chips (*Quercus alba*, *Quercus petraea*, *Quercus pyrenaica*, *Castanea sativa*, *Prunus avium*) used in ageing processes and find out which compounds could contribute to spirits and wines during its maturation in order to optimize a methodology that could be applied for the analysis of not only the wood chips but also the staves of the wooden barrels used in cooperage. In order to characterize the aromatic profile of each type of wood, the volatile composition was studied by DTD-GC-MS (Direct Thermal Desorption-Gas Chromatography-Mass Spectrometry). Moreover, grounded wood chips were extracted by 50% hydroalcoholic solution in order to determine which compounds could be released into the spirit or the wine. The extracts were characterized by GC-MS (Gas Chromatography-Mass Spectrometry), SBSE-GC-MS (Stir-bar Sorptive Extraction–Gas Chromatography-Mass Spectrometry), UHPLC (Ultra-High-Performance Liquid Chromatography) and TPI (Total Phenolic Index).

## 2. Materials and Methods

### 2.1. Wood Samples

Five different kinds of wood were studied: American oak (*Quercus alba*), French oak (*Quercus petraea*) and Spanish oak (*Quercus pyrenaica*) with a medium toasting level; and Cherry (*Prunus avium*) and Chestnut (*Castanea sativa*) wood without toasting.

Samples were in the form of wood chips (5–15 mm length × 5–10 mm width × 2 mm thickness) and 100 g of each wood were ground to a 0.25 µm grain size powder with an ultra-centrifugal mill ZM 200 (Retsch GmbH, Haan, Germany) before analysis and extraction. Chips were obtained from Roble Enológico, S.L. (Cantabria, Spain). For the optimization of the DTD-GC-MS methodology a mixture of equal parts of the five studied wood chips was used.

### 2.2. Reagents

The rectified wine distillate at 96% vol. used in this study was supplied by Bodegas Fundador, S.L.U. (Jerez de la Frontera, Spain). For the wood extraction experiments, it was diluted with ultrapure water from EMD Millipore (Bedford, MA, USA) until it reached 50% vol. of alcoholic strength.

UHPLC grade acetonitrile from Panreac (Barcelona, Spain) and acetic acid from Merck (Darmstadt Germany) were used to prepare the UHPLC phases. Standards for calibration were purchased from Sigma-Aldrich (Saint Louis, MO, USA). Ultrapure water from EMD Millipore (Bedford, MA, USA) was used to prepare the chromatography phases, reagents and standards for calibration.

4-methyl-2-pentanol (Sigma-Aldrich, Steinheim, Germany) was employed as an internal standard in SBSE-GC-MS and DTD-GC-MS analysis.

### 2.3. Analysis of Volatile Compounds of Wood Powder by DTD-GC-MS

This study was based on two factorial experiments: a factorial design of 2^4^ was chosen to determine the most influential parameters of the direct thermal desorption process and a 3^2^ experiment was carried out to establish the optimum values of these parameters. The final conditions considered to be optimal were the following: heating 10 mg of the sample at 250 °C during 7 min, desorbing the sample at 250 °C during 6 min and transferring the desorbed compounds to the line at 1:10 ratio split. The Statgraphics Statistical Computer Package “Statgraphics Centurion 18.0” was used for data treatment.

After the method optimization, the analysis of volatile compounds of wood powder were carried out by DTD-GC-MS. An amount of 10 mg of ground wood was placed together with 5 µL of a solution of 4-methyl-2-pentanol (303 mg L^−1^ in an ethanol-water solution at 40% of alcohol) in the desorption tube, plugged at both ends with silanized glass wool. The desorption tube was heated to 250 °C for 7 min. The volatile compounds were desorbed in a stream of helium and collected into a cold trap (−15 °C). The desorption was carried out at 250 °C during 6 min and the volatile compounds were transferred (split 1:10) to the chromatographic column through a line heated to 225 °C. The experiments were carried out in a GCMS-TQ8040 Shimadzu gas chromatograph with mass detection (Shimadzu, Kyoto, Japan) equipped with a DB-Wax capillary column (J&W Scientific, Folsom, CA, USA), 60 m × 0.25 mm I.D., with a 0.25 µm coating. The chromatographic conditions were the same as the ones used previously. Samples were analyzed in duplicate. The relative area of each compound was obtained by measuring the area of the chromatographic signal produced by largest mass fragment (base peak) with respect to that of the internal standard, 4-methyl-2-pentanol. The results were expressed in relative area values.

### 2.4. Ultrasound-Assisted Extraction from Wood Powder

A total 1.1 g of wood powder was extracted with 200 mL of rectified wine distillate at 96% vol./water (1:1) hydroalcoholic solution at 40 °C during 2.5 h using an ultrasonic bath system (JP Selecta, S.A., Abrera, Spain) with 38.5 W L^−1^ power as accelerating energy of the extraction process. The powder was washed with 50 mL of the same mixture used before. The extract was centrifuged at 5000 rpm during 5 min and transferred to a 250 mL volumetric flask. The extracts were used for the SBSE-GC-MS, GC-MS and UHPLC determinations. Each wood extraction was carried out in duplicate. The analysis of each extraction was also carried out in duplicate. Samples were stored in darkness under refrigeration.

### 2.5. Analysis of Volatile Compounds of Wood Extracts by SBSE-GC-MS and GC-MS

Volatile compounds of wood extracts were analyzed by SBSE-GC-MS and GC-MS techniques. PDMS commercial stir bars (10 mm length × 0.5 mm film thickness) provided by Gerstel (Mülheim a/d Ruhr, Germany) were used for the extractions. The procedure stablished in previous investigations of our research group was followed [24]: a volume of 35 mL of sample was placed in an Erlenmeyer flask and was diluted 1:1 (*v*/*v*) with ultrapure water. Then, 140 µL of a solution of 4-methyl-2-pentanol (2.3056 g L^−1^ in an ethanol-water solution at 50% of alcohol) was added as an internal standard. Once the stir bar was added, the flask was placed on a 15-position magnetic stirrer (Mülheim a/d Ruhr, Germany) under agitation during 100 min at 1100 rpm at 25 °C. Finally, the stir bar was removed and washed and transferred into a thermal desorption glass where the thermal desorption was carried out. A commercial TDU (thermal desorption unit, Gerstel) with a programmed temperature vaporisation injector CIS-4 (cooled injection system, Gerstel) was used to carried out the thermal desorption of the coated stir bars. The desorption temperature was programmed from 40 to 300 °C (held for 10 min) at 60 °C min^−1^ under a helium flow (75 mL min^−1^) and the desorbed compounds were cryofocused in the CIS-4 system with liquid nitrogen at −140 °C. Finally, the CIS-4 was programmed from −140 °C to 300 °C (held for 5 min) at 10 °C/s for analysis by GC-MS. An Agilent 6890 GC-5973N MS system (Agilent, Little Falls, DE, USA), equipped with a DB-Wax capillary column (J&W Scientific, Folsom, CA, USA), 60 m × 0.25 mm I.D., with a 0.25 µm coating, was used to carried out the capillary GC-MS analyses in the electron impact mode. Helium was used as the carrier gas at a flow rate of 1.0 mL min^−1^. The GC oven was programmed as follows: held at 35 °C for 10 min, then ramped at 5 °C min^−1^ to 100 °C. Then it was raised to 210 °C at 3 °C min^−1^ and held for 40 min. The mass detector operated in EI+ mode at 70 eV in a range from 30 to 400 amu. The identification of the compounds was carried out by analogy with mass spectra held in the Wiley Library (*Wiley Registry of Mass Spectral Data*, 7th Edition, 2000) and confirmed by retention indices of standards. The relative area of each compound was obtained by measuring the area of the chromatographic signal produced by largest mass fragment (base peak) with respect to that of the internal standard, 4-methyl-2-pentanol. Seventeen compounds were identified: ethyl butyrate, isoamyl acetate, limonene, ethyl caprylate, ethyl caprate, isopentyl octanoate, ethyl 2-phenylacetate, ethyl laureate, caprylic acid, ethyl myristate, capric acid, ethyl palmitate, ethyl 9-hexadecenoate, ethyl stearate, lauric acid, myristic acid and pentadecanoic acid. The results were expressed in relative area values.

Regarding the GC-MS analysis, the followed temperature program was the same as in the SBSE-GC-MS. The experiments were carried out in a GCMS-TQ8040 Shimadzu gas chromatograph with mass detection (Shimadzu, Kyoto, Japan) equipped with a DB-Wax capillary column (J&W Scientific, Folsom, CA, USA), 60 m × 0.25 mm I.D., with a 0.25 µm coating (the same column as the SBSE-GC-MS equipment).

### 2.6. Analysis of Phenolic Compounds and Furfurals in the Wood Extracts

Nine phenolic compounds (gallic acid, ellagic acid, caffeic acid, vanillic acid, vanillin, syringic acid, syringaldehyde, sinapaldehyde and coniferylaldehyde) and two furanic aldehydes (furfural and 5-hydroxymethylfurfural) were identified and quantified by UHPLC. Two eluents were used: a phase that consisted of 3% acetonitrile, 2% acetic acid and 95% ultrapure water, and B phase that consisted of 85% acetonitrile, 2% acetic acid, and 13% ultrapure water The method stablished in previous investigations of our research group was followed [25] for these analysis: 0 min, 100% A; 3 min, 90% A; 4 min, 90% A; 6.5 min, 25% A with a flow rate of 0.7 mL min^−1^ and a column temperature of 47 °C. The injection volume was 2.5 µL. The column was washed with 100% B for 3 min and equilibrated with 100% A for 3 min. Then, 0.22 µm nylon membranes were used to filtered samples and standards. The detection by UV absorption was conducted by scanning between 250 and 400 nm, with a resolution of 1.2 nm. The comparison of retention times and UV-Vis spectra of the peaks in samples with those previously obtained by the injection of standards allows the identification of each compounds. Samples and standards were injected in duplicate. The results were expressed in mg of compound per liter of sample.

### 2.7. Total Polyphenol Index in the Wood Extracts

Total Polyphenol Index (TPI) of the wood extracts was determined by the measure of the absorbance at 280 nm. Samples were measured directly or diluted with ultrapure water where necessary. A Lambda 25 spectrophotometer (Perkin Elmer, Boston, MA, USA) was used for the analysis. The calibration curve was prepared with gallic acid solutions ranging from 0 to 50 mg L^−1^. A glass cell with a 10 mm optical path was used. Sample measurements were carried out in duplicate. The results are expressed in mg of gallic acid equivalent (GAE) per litre of sample.

### 2.8. Statistical Analysis

The Statgraphics 18 software package (Statgraphics Technologies, Inc., The Plains, VA, USA) was employed for factorial design experiments and ANOVA. Microsoft Excel 2016 (Microsoft Corp., Redmond, WA, USA) was employed for other statistical parameters.

## 3. Results and Discussions

### 3.1. DTD-GC-MS Condition Optimization

Heating temperature, heating time, desorption temperature and desorption time of the direct thermal desorption process were evaluated to achieve the best overall analytical conditions. No references related with wood and the volatile compounds that it could bring to spirits and wines determined by this method were found in bibliography and, therefore, it had to be optimized.

To optimize the direct thermal desorption conditions, we chose a sequential exploration of the response, which was carried out in two stages. In the first stage, a factorial design of 2^4^ was chosen to analyze the influence of heating temperature, heating time, desorption temperature and desorption time using a mixture of all the wood types studied, as described in Section 2.1, in order to consider all the compounds that could be present in the wood samples. In the second stage, a factorial design of 3^2^ was chosen to optimize the heating temperature and heating time.

#### 3.1.1. Screening by a 2^4^ Factorial Design

The values corresponding to the low (−) and high (+) levels for each factor are shown in Table 1. The design involved sixteen experiments in duplicate. Total area values and chromatographic peak number of each experiment evaluated in the 2^4^ factorial design are shown in Table 2. The data obtained for the heating temperature, heating time, desorption temperature and desorption time were evaluated by ANOVA at the 0.05 significance level (Table 3).

**Figure 1:**
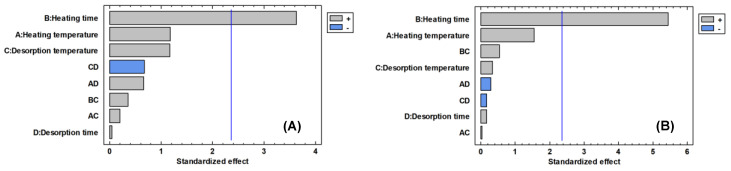
Pareto chart of main effects in the 2^4^ factorial design for number of chromatographic peaks (**A**) and total area (**B**).

**Figure 2:**
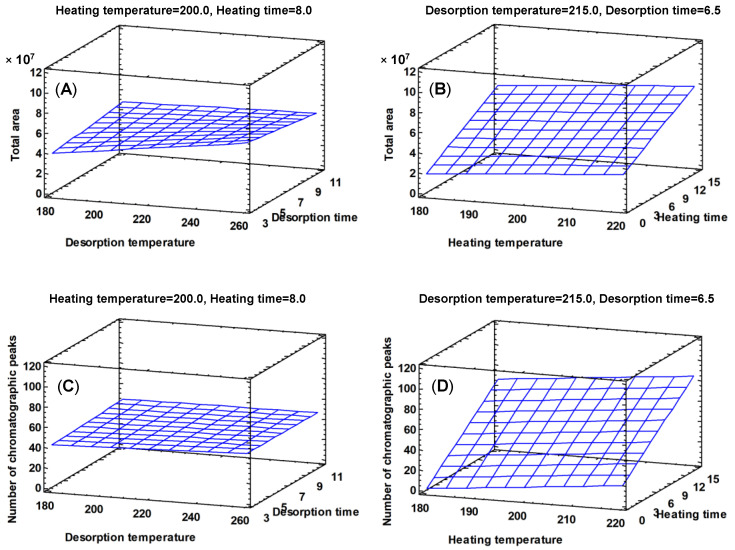
Interaction heating time–heating temperature. Estimated response surface for total chromatographic area (**A**) and for the number of chromatographic peaks (**C**). Interaction desorption time–desorption temperature. Estimated response surface for total chromatographic area (**B**) and for the number of chromatographic peaks (**D**).

Parameters related with the heating process had a significant positive influence on the total area and the number of chromatographic peaks, appearing statistically as the most influential effect (Figure 1). The effect of the parameters heating time and heating temperature is positive for the two variables considered, that is, high temperature levels and high heating time produce the extraction of larger amounts of volatile compounds (Figure 2), being the heating time the only parameter that presents a significant effect (*p* value < 0.05, Table 3), both for the number of peaks and for the total area. The heating temperature is the next parameter that most affects the variables considered, although its effect is not significant (*p* value > 0.05, Table 3). As the heating time and the heating temperature increase (15 min and 220 °C, respectively), the response obtained in both variables is greater (Figure 2).

The parameters related with the desorption process, temperature desorption and time desorption do not show a significant influence on the total area or the number of chromatographic peaks (*p* value > 0.05, Table 3). However, the desorption at 250 °C showed better results than the desorption at 180 °C, being selected as the optimal value for the following analysis (Figure 2). No differences between the high and low level of the desorption time were found, so an average value (6 min) was selected.

Heating parameters turned out to be the most influential ones in the direct thermal desorption process. For these analysis, 20 mg of a mixture of the five woods was used. Some chromatographic peaks were saturated and so, for the optimized method experiments, the sample amount employed was lower than before in the following factorial design.

In summary, the best conditions obtained in this first optimization study were the following: heating time, 15 min; heating temperature, 220 °C; desorption time, 6 min; and desorption temperature, 250 °C.

#### 3.1.2. Optimization by a 3^2^ Factorial Design

In order to optimize the parameters of the direct thermal desorption method, the most influent variables resulting from the first factorial design were studied. Three levels of heating temperature and heating time were established. The design involved nine experiments in duplicate. The values corresponding to the low (−) and high (+) levels for each factor are shown in Table 4.

After the results obtained in the 2^4^ factorial design experiments, the desorption conditions established for the analysis were the following: 6 min and 250 °C. A total of 10 mg of the mixture sample was used in the study. Total area values and chromatographic peak number of each experiment evaluated in the 3^2^ factorial design were shown in Table 5. The data obtained for the heating temperature and the heating time were evaluated by ANOVA at the 5% significance level (Table 6).

**Figure 3:**
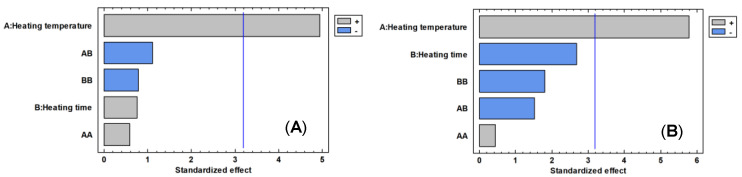
Pareto chart of main effects in the 3^2^ factorial design for the number of chromatographic peaks (**A**) and total area (**B**).

**Figure 4:**
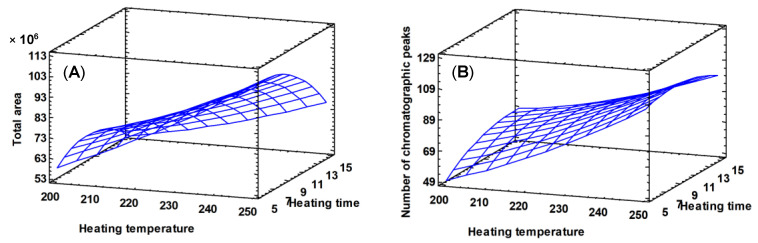
Interaction heating time–heating temperature. Estimated response surface for total chromatographic area (**A**) and for the number of chromatographic peaks (**B**).

Heating temperature had a significant positive influence on the total area and the number of chromatographic peaks, appearing as the statistically main effect (Figure 3). The effect of heating time and heating temperature is positive for the two variables considered, that is, high temperature levels produce the extraction of larger amounts of volatile compounds as the heating time value is between 5 and 10 min (Figure 4). The heating temperature is the only parameter that presents a significant effect (*p* value < 0.05, Table 6), both for the number of peaks and for the total area. The heating temperature does not significantly affect the total area or the number of chromatographic peaks (*p* value > 0.05, Table 6). However, the heating time range from 5 to 10 min showed the best results, and so, an average value (7 min) was selected as the optimum value.

Taking into account all the results obtained, the final direct thermal desorption conditions considered to be optimal were as follows: heating 10 mg of the sample at 250 °C during 7 min and desorbing the sample at 250 °C during 6 min.

### 3.2. Analysis of Volatile Compounds of Wood Powder by DTD-GC-MS

Five different types of wood (American oak, Spanish oak, French oak, Chestnut and Cherry) were analyzed (in duplicate) employing the DTD-GC-MS method optimized. For this analysis, the wood chips were grounded to a 0.25 µm grain size. All the factorial design experiments were carried out in a splitless mode. High peak densities were obtained in all of them. In order to avoid detector saturation during the analysis of the real samples, they were injected in split mode. Different split ratios were tested 1:30, 1:20, 1:10 and 1:5. The split ratio 1:10 showed the best results.

The amount of the volatile compounds detected in each type of wood has been obtained by means of the relative integration with respect to the internal standard, 4-methyl-2-pentanol (Table 7). The results were evaluated by ANOVA at the 5% significance level (Table 7).

**Figure 5:**
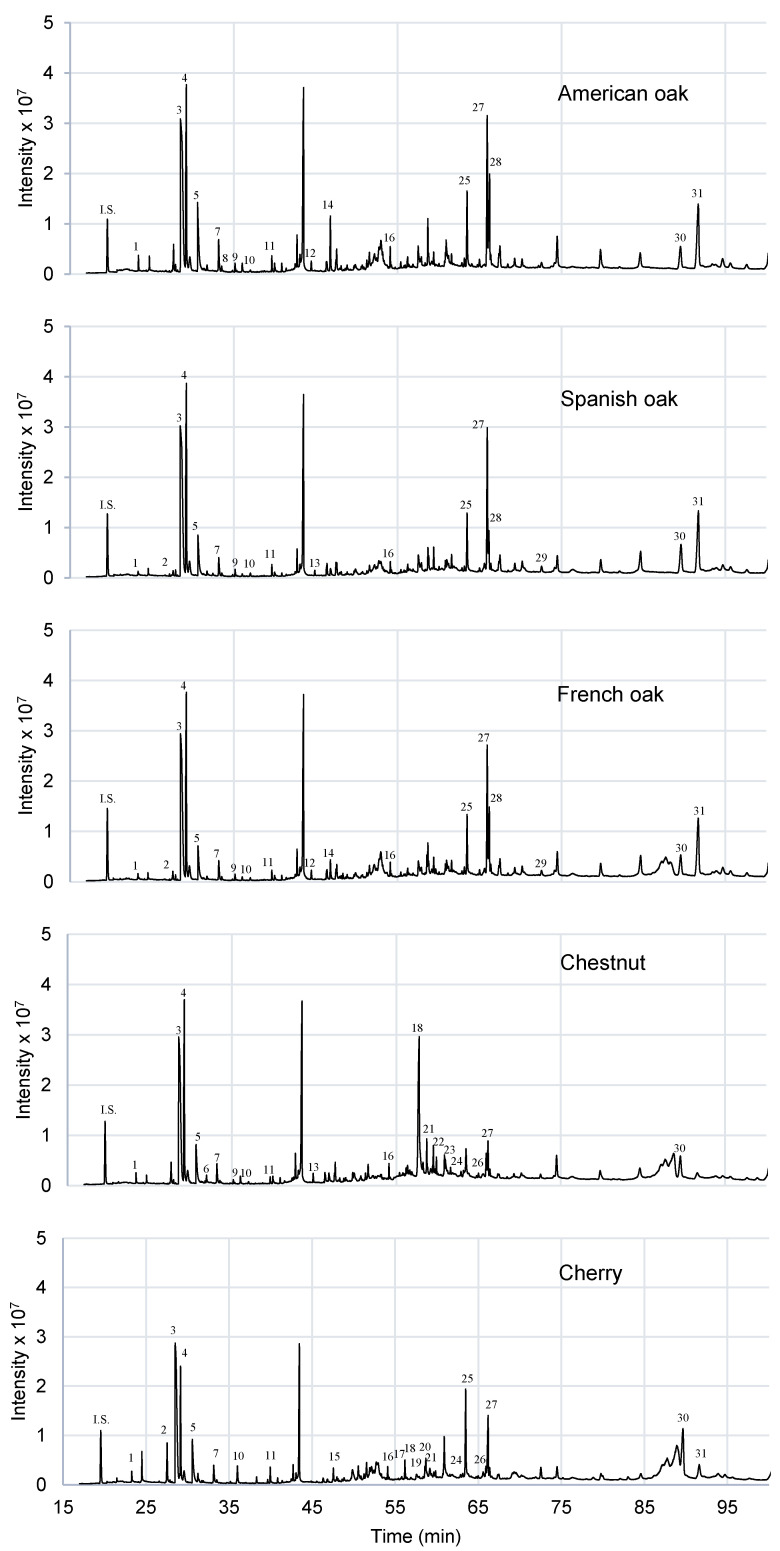
DTD-GC-MS chromatogram of the hydroalcoholic wood extracts. IS: Internal Standard (4-methyl-2-pentanol). The key for the compounds is in Table 7.

As expected, many similarities were found regarding the volatile composition of three oak woods studied. As can be seen in Figure 5, their aroma profile is very similar. However, there are some differences, as the levels of formic acid, acrylic acid and furanone that are significantly different, at 5% of significance level, in American oak with respect to Spanish and French oaks (Table 7). There are significant differences between the amount of vanillin and syringaldehyde between American and French oak, but there are none as compared to Spanish oak (Table 7). These compounds could be affected by the toasting level of the chip wood. All the studied oaks have a medium toasting level. Acetic acid, furfural, formic acid, 5-methylfurfural, vanillin and syringaldehyde are the most abundant components [20,23,26,27]. Many of them are generated during the heat treatment processes during the toasting of the chips. There are compounds as whiskey lactones that are only identified in American and French oaks. According to the literature, these compounds are commonly present in oak wood, being more abundant in the *Quercus alba* species [20,22]. 2-phenylethanol is only detected in Spanish oak and chestnut. 4-cyclopentene-1,3-dione is only detected in American oak. Myristic acid is detected in French and Spanish oak [5] but it is not detected in American oak.

In Figure 5, chestnut and cherry show a slightly different aromatic profile both between them and with respect to the oak types studied. Most of the detected compounds were in significant different amounts with respect the other woods studied (Table 7). However, they contain many compounds present in oak wood too, like acetic acid, furfural, formic acid, 5-methylfurfural and palmitic acid [20,22,26], and their content is not significantly different among woods at 5% significance level (Table 7), except for formic acid content. It should be noted that the cherry and chestnut wood studied in both cases is an untoasted wood; however, they are also rich in compounds that come from the toasting of the wood, such as furfurals. This is due to the prior heating of the sample as part of the analysis method. The peak profile during the first 40 min of the analysis is very similar for all the wood chips studied. Cherry is the wood that has the most different volatile composition profile. 2,3-butanediol, glycerin and ethyl hydrogen succinate are only present in chestnut. This wood is similar to the studied oak woods, but also shares some similarities with cherry wood. Compounds as 2-phenylethanol or acrylic acid are detected in oak and chestnut wood, but are not present in cherry wood. On the other hand, compounds as trans-isoeugenol, 2,3-dihydrobenzofuran, benzoic acid and methoxyeugenol are present in chestnut and cherry wood but not in oak wood. According to the literature, methoxyeugenol is present in significant levels in cherry and chestnut wood, it could be present in oak wood too but, in this case it was not detected [5,28]. Cherry wood has the most particular aroma profile. Cyclopropyl carbinol, pyranone [20], levulinic acid and p-acetylacetophenone are only detected in this wood. There are some compounds as 5-HMF, ethriol and syringaldehyde that are detected also in oak wood, but they are not in chestnut wood. Therefore, there are compounds or profiles that are characteristics of each type of wood studied, thus being able to be targets to identify each species in DTD-GC-MS analysis.

All the mentioned studies analyzed the volatile compounds through a previous hydroalcoholic extraction of the wood; the novelty of this work is the direct analysis of wood aromas by DTD-GC-MS. Although there are studies in oak wood that work with DTD-GC-MS (but using a solid support to trap the volatile compounds) [29], in the rest of cited references, the woods have not been studied in this way. The previous direct thermal desorption stage could alter the sample, since heating could increase the toasting level of wood sample studied and increase the concentration of those compounds that are related to this process. However, there is not a loss of information, as could happen in extraction processes, since the aromatic profile is measured directly.

### 3.3. Analysis of Volatile Compounds of Wood Extracts by SBSE-GC-MS and GC-MS

An analysis on hydroalcoholic wood extracts was carried out to compare the compounds that are present in the wood detected by the analysis by DTD-GC-MS with those volatile compounds that could be transferred to the spirit or the wine during their ageing through the wood chips. In order to determine if they could be detected in aged alcoholic beverages, an hydroalcoholic ultrasound assisted extraction was carried out.

As regards the volatile composition of wood extracts, a low amount of the compounds was found in the samples. Relative area values of volatile compounds determined by SBSE-GC-MS of wood extracts are shown in Table 8. The aromatic profile is different in all the studied wood but the amount of each detected compound is very low. In order to complement this information, the samples were analyzed by GC-MS. However, any compounds were not detected with this technique.

Attending to the results, shown in Table 8, wood related compounds were not detected. As regards the relative area values of the compounds present in the extractant (1:1 hydroalcoholic mixture of rectified wine distillate at 96% vol. and water solution), similarities with the relative area values of wood extracts were found. The relative area values obtained were evaluated by ANOVA at the 5% significance level (Table 8). As it can be seen, most of them are not significantly different, which means that the compounds detected are not influenced by wood. It seems that it only contributes to trace levels of them. According to the literature, there are fatty acids as caprylic acid, myristic acid or palmitic acid present in wood composition [30,31,32]. The contribution of these compounds to the extracts and their respective esters could be due to the extraction procedure, in which the wood powder was extracted with an hydroalcoholic solution under 40 °C. The esterification of the fatty acids in the presence of ethanol at this temperature resulting in the corresponding esters as ethyl caprylate, ethyl myristate, ethyl palmitate or ethyl laureate could take place during the extraction process. This fact could explain the increase of the ethyl laureate and myristic acid in all the wood extracts studied, ethyl caprylate in chestnut and cherry wood extracts and ethyl palmitate in the American oak extract. The only compound detected in both analysis (DTD-GC-MS and SBSE-GC-MS) was myristic acid.

In the direct analysis of the wood powder by DTD-GC-MS, numerous volatile compounds were identified, which make it a very interesting technique. These compounds are transferred from the wood to the spirit or the wine during their ageing, modifying its sensorial profile. During ultrasound-assisted extractions, these compounds were extracted by the hydroalcoholic mixture used. However, once the hydroalcoholic extracts were analyzed, no volatile compounds were detected by GC-MS and very few compounds and at very low levels were detected by SBSE-GC-MS. Therefore, there is a loss of information regarding the analysis of volatile compounds once the ultrasound-assisted extraction is performed. However, the hydroalcoholic extracts were useful to characterize the phenolic compounds that wood could contribute to spirits and wines and to complete the aromatic profile of the woods studied. The majority of the compounds identified in SBSE-GC-MS analysis (Table 8) are characteristic of wines, wine spirits or brandies, so their presence in the wood extracts studied could be also due to the origin of the extractant used, that has a part of a grape derived alcoholic beverage.

### 3.4. Phenolic Composition of the Wood Extracts and Total Polyphenol Index

The TPI data of the studied samples, expressed in mg of equivalent gallic acid (GAE) per litre, are shown in Table 9. Of all the woods studied, Spanish oak released the highest amount of phenolic compounds into the alcoholic beverage. The lowest TPI values of oak were found in American oak wood. The TPI values for cherry wood (without toasting) are between medium toasted French and American wood. Chestnut (without toasting) has the lowest composition in phenolic compounds of all those studied. The results of the one-way analysis of the variance (ANOVA) proved that all the wood extracts are statistically different, with a probability of 95%.

The content in low molecular weight phenolic compounds determined by means of UHPLC in the wood extracts, expressed in mg L^−1^, is also shown in Table 9. As regards the phenolic acids studied, gallic acid, vanillic acid, caffeic acid, syringic acid and ellagic acid were found. Gallic and ellagic acids come from the hydrolysis of gallotannins and ellagitannins under an acidic environment [28]. The oxidation and hydrolysis of the compounds derived from the degradation of lignin is the origin of vanillic and syringic acids [16,33]. A significant amount of phenolic aldehydes (p-hydroxybenzaldehyde, vanillin, syringaldehyde, coniferylaldehyde and sinapaldehyde) was found in some of the samples studied (Table 9). Their origin is in the thermal degradation of lignin [14,16,33], a process that takes place during the manufacturing of the barrel due to the toasting of the wood and its thermal treatments [10]. 5-hydroxymethylfurfural and furfural have been detected in significant amounts in some of the samples studied (Table 9). The presence of furfural is due to the heating of the pentoses, while 5-hydroxymethylfurfural has its origin in the thermal degradation of the glucose and cellulose. Their presence depends on the toasting of the wood [10].

**Figure 6:**
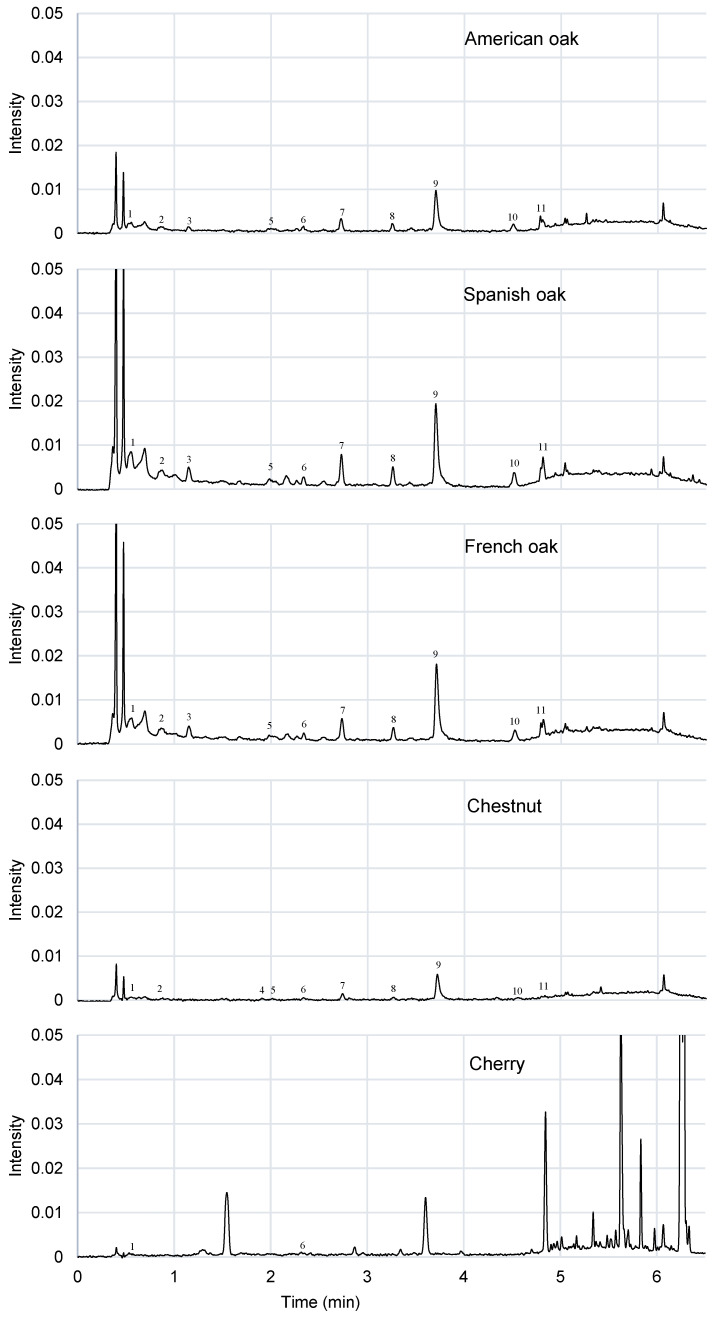
Ultra-High-Performance Liquid Chromatography (UHPLC) chromatogram comparison of the hydroalcoholic wood extracts at 280 nm. The key for the compounds is in Table 9.

Phenolic composition is very similar in American, Spanish and French oak (Figure 6). The same compounds were found in the hydroalcoholic extracts of all of them, however their proportion was not the same. Gallic acid, ellagic acid, vanillic acid, vanillin, syringaldehyde, coniferylaldehyde and sinapaldehyde are the most abundant compounds detected in the oak wood extracts. Spanish oak has the highest amount of phenolic compounds, while American oak has the lowest quantity. The amount of phenolic compounds present in each wood is influenced by the origin and the heat treatment of the wood during the manufacturing of the barrel or chips [10]. During the ageing period, wood characteristics as porosity affect the extraction of phenolic and volatile compounds. Spanish and French oak are more porous than American oak, and this has a positive influence during the extraction process.

All the compounds detected in oak wood, except furfural, were also found in Chestnut extracts. This wood was untreated, without toasting treatment, so it explains the absence of this compound. However, due to its porosity, a great amount of phenolic compounds was found in the hydroalcoholic extracts studied. Chestnut wood has high levels of gallic acid [34], in this case the wood studied was not toasted, so the level of this compound is lower than expected.

As regards the cherry wood chromatogram, a high level of phenolic compounds was detected. There are many signals at the end of the chromatogram indicating that, according to their retention time, these are low-polar compounds. According to the literature, they could be flavonoid-type compounds [23] as (+)-catechin [35], taxifolin [36], naringenin [27], aromadendrin [37] or kaempferol [37], that are very common in cherry wood. Flavonoids were only detected in cherry wood (Figure 6), what makes its aromatic profile very interesting. It would be interesting to be able to identify these compounds in the future, because they make cherry wood an alternative material for the ageing of spirits and wines and to obtain different sensorial profiles from oak or chestnut. There are other compounds, as vanillin, vanillic acid, syringaldehyde, sinapaldehyde or coniferylaldehyde that come from lignin degradation and are also present in cherry wood [23], but their presence is higher when the wood is toasted. In this sample, these compounds are at trace level, below the limit of quantification, and thus, they could not be quantified.

In summary, a similar profile has been observed in the three types of oak studied. Oak wood is rich in ellagitannins and low molecular weight acids and aldehydes. No flavonoids have been detected in any of them [26]. Besides being from the same family, the three oak wood chips analyzed were toasted. During the toasting process, wood increases its concentration of compounds derived from lignin, and a different reduction between phenolic profiles was observed for the different woods [26]. On the other hand, untoasted chestnut chip wood was studied. This wood is slightly similar to oak wood; it is also rich in ellagitannins and low molecular weight acids and aldehydes. In this wood, there is also an absence of flavonoids [26]. However, as this wood was untoasted, it has a low concentration in compounds derived from lignin. As regards cherry wood, a phenolic profile totally different from the rest of the woods was observed. This wood is rich in flavonoid-type compounds and has a certain deficiency in ellagitannins and derivatives. It was also untoasted, so a low concentration of compounds derived from lignin was found. Cherry wood has a specific profile of low molecular weight compounds [18,23,26,38].

Furfural, hydroxymethylfurfural, vanillin and syringaldehyde were also detected in DTD-GC-MS analysis of wood powder. Furfural and hydroxymethylfurfural levels are higher in DTD-GC-MS experiments than in UHPLC analysis. In DTD-GC-MS, during the prior direct thermal desorption process there is a heating of the sample, reaching high temperatures that affect it, and these compounds are related with the toasting level of the wood. Vanillin and syringaldehyde are also found in a higher amount in DTD-GC-MS, except for chestnut wood. They are compounds that come from lignin thermal degradation, being also affected by high temperatures. During direct thermal desorption, wood is toasted, which produces an alteration of the initial sample that generates differences between the level of the compounds determined by both analyses.

The phenolic compound content determined by UHPLC was evaluated by ANOVA at the 5% significance level (Table 9). As can be seen, most of them are significantly different, which means that the phenolic profile is characteristic of each wood. Although there are similarities in the presence of some compounds in the three oak woods studied or with chestnut wood, the proportion of them in each wood is different. Only a few similarities were found regarding syringic acid (no significant differences between American and French oak at the 5% significance level were found), vanillic acid (no significant differences between Spanish oak and chestnut at the 5% significance level were found) and hydroxymethylfurfural (no significant differences between Spanish and French oak at the 5% significance level were found).

## 4. Conclusions

The conditions for the DTD-GC-MS of wood samples were optimized and a method for the direct characterization of wood chips studied was established. The optimal direct thermal desorption conditions determined were the following: heating 10 mg of the sample at 250 °C during 7 min, desorbing the sample at 250 °C during 6 min and transferring the desorbed compounds to the line at 1:10 ratio split.

In this study, five different wood chips were characterized. The characterization of their aroma profile by DTD-GC-MS was carried out and different profiles of each wood were determined. Compounds as acetic acid, furfural, 5-methyfurfural and palmitic acid were found in all types of wood studied. However, compounds as whiskey lactones (American and French oak), 4-cyclopentene-1,3-dione (American oak), 2,3-butanediol (Chestnut), glycerin (Chestnut), ethyl hydrogen succinate (Chestnut), cyclopropyl carbinol (Cherry), pyranone (Cherry), levulinic acid (Cherry) or p-acetylacetophenone (Cherry) are only present in certain types of wood, which make them interesting target compounds to identify these woods. Similar volatile compounds were detected in all the woods studied during the first 40 min of the analysis. However, the aromatic profile is totally different from one wood to another at the end of the chromatogram. These differences allow them to be distinguished. This method is a potential technique to identify aromas in wood that, in addition, allows to differentiate between different types of wood.

To analyze the aromatic profile of the hydroalcoholic wood extracts, SBSE-GC-MS was employed. The differentiation of the woods was not possible by this technique due to the similarity of all the chromatograms obtained between them and the extractant used. However, the phenolic profile of wood extracts, determined by UHPLC, allowed this differentiation. There were differences between the phenolic profile of oak woods, and chestnut and cherry wood extracts. Cherry wood extract has the most particular phenolic profile of the wood extracts studied.

Direct determination of wood aromas is possible due to the direct thermal desorption. This technique allows the analysis of wood in solid state, without any type of previous treatment, except grinding. DTD-GC-MS analysis allows the determination of the aromatic profile, without any loss of information. There are other techniques that involve a previous treatment of the sample, such as extraction, where some aromatic compounds may be lost. Furthermore, the information obtained by DTD-GC-MS was characteristic of each type of wood, allowing its differentiation.

This work established and optimized a novel method for the characterization of wood chips in a direct way, and for the characterization of their extractable compounds, allowing its application to other types of samples as barrel staves. This is an interesting strategy that could be applied, not only for the analysis of wood chips, but also for wooden barrels used during the ageing process of spirits and wines.

## Figures and Tables

**Table 1:** Levels of the 2^4^ factorial design.

Factor	Low (−)	High (+)
Heating temperature (°C)	180.0	220.0
Heating time (min)	1.0	15.0
Desorption temperature (°C)	180.0	250.0
Desorption time (min)	3.0	10.0

**Table 2:** Conditions, total area and number of chromatographic peaks obtained in each experiment of the 2^4^ factorial design.

Experiment	Heating Temperature (°C)	Heating Time (min)	Desorption Temperature (°C)	Desorption Time (min)	Total Area	Number of Peaks
1.1	180	15	180	10	45,266,253	57
1.2	180	15	180	3	45,342,152	40
1.3	180	15	250	10	52,191,184	63
1.4	180	1	250	10	20,703,659	16
1.5	220	1	180	10	24,144,870	6
1.6	220	15	250	3	97,177,735	110
1.7	180	1	180	10	41,301,225	21
1.8	180	1	180	3	19,116,371	22
1.9	220	1	180	3	11,964,472	14
1.10	220	15	250	10	112,801,779	100
1.11	180	1	250	3	61,846,897	22
1.12	220	1	250	3	23,463,052	5
1.13	220	15	180	3	80,695,112	91
1.14	220	1	250	10	29,182,299	11
1.15	180	15	250	3	62,341,956	53
1.16	220	15	180	10	80,828,868	98

**Table 3:** Main effects and interactions in the 2^4^ factorial design for the number of chromatographic peaks and total area.

Effect	No. of Chromatographic Peaks		Total Area	
	F Ratio	*p* Value	F Ratio	*p* Value
A: Heating temperature	2.41	0.1643	1.39	0.2763
B: Heating time	29.73	0.0010	13.19	0.0084
C: Desorption temperature	0.12	0.7428	1.37	0.2806
D: Desorption time	0.03	0.8734	0.00	0.9638
AC	0.00	0.9746	0.04	0.8477
AD	0.08	0.7910	0.44	0.5294
BC	0.29	0.6061	0.13	0.7331
CD	0.03	0.8734	0.46	0.5197

**Table 4:** Levels of the 3^2^ factorial design.

Factor	Low (−)	High (+)
Heating temperature (°C)	200.0	250.0
Heating time (min)	5.0	15.0

**Table 5:** Conditions, total area and number of chromatographic peaks obtained in each experiment of the 3^2^ factorial design.

Experiment	Heating Temperature (°C)	Heating Time (min)	Total Area	Number of Peaks
2.1	220	10	83,670,835	91
2.2	200	15	56,175,969	76
2.3	250	10	96,680,416	110
2.4	250	5	103,356,194	102
2.5	220	15	54,743,149	70
2.6	220	5	84,269,330	74
2.7	200	5	54,598,381	52
2.8	250	15	82,280,240	102
2.9	200	10	65,838,463	55

**Table 6:** Main effects and interactions in the 3^2^ factorial design for number of chromatographic peaks and total area.

Effect	No. of Chromatographic Peaks		Total Area	
	F Ratio	*p* Value	F Ratio	*p* Value
A: Heating temperature	24.47	0.0159	33.52	0.0103
B: Heating time	0.57	0.5050	7.21	0.0747
AA	0.35	0.5975	0.18	0.6969
AB	1.23	0.3480	2.31	0.2259
BB	0.62	0.4898	3.24	0.1695

**Table 7:** Relative area values of volatile compounds determined by DTD-GC-MS of wood powder.

Compound	American Oak	Spanish Oak	French Oak	Chestnut	Cherry
Acetol **(1)**	0.112 ± 0.025 ^a^	0.045 ± 0.003 ^a^	0.046 ± 0.008 ^a^	0.054 ± 0.011 ^a^	0.501 ± 0.104 ^b^
2-*O*-cyclobutyl 1-*O*-octadecyl oxalate **(2)**	n.d.	0.020 ± 0.028	0.089 ± 0.014	n.d.	0.372 ± 0.526
Acetic acid **(3)**	7.158 ± 1.123	5.906 ± 0.453	5.079 ± 0.639	4.779 ± 0.799	4.673 ± 2.868
Furfural **(4)**	2.813 ± 0.425	2.574 ± 0.245	2.497 ± 0.364	2.213 ± 0.327	1.976 ± 0.241
Formic acid **(5)**	1.653 ± 0.276 ^a^	0.825 ± 0.043 ^b^	0.687 ± 0.112 ^b^	0.714 ± 0.118 ^b^	1.191 ± 0.520 ^a,b^
2,3-butanediol **(6)**	n.d.	n.d.	n.d.	0.082 ± 0.013	n.d.
5-methylfurfural **(7)**	0.466 ± 0.118	0.231 ± 0.027	0.277 ± 0.041	0.242 ± 0.021	0.237 ± 0.294
4-cyclopentene-1,3-dione **(8)**	0.054 ± 0.016	n.d.	n.d.	n.d.	n.d.
Acrylic acid **(9)**	0.103 ± 0.020 ^a^	0.069 ± 0.006 ^b^	0.059 ± 0.008 ^b,c^	0.040 ± 0.004 ^c^	n.d.
Furfuryl alcohol **(10)**	0.118 ± 0.020 ^a^	0.028 ± 0.007 ^a^	0.038 ± 0.008 ^a^	0.073 ± 0.012 ^a^	0.390 ± 0.087 ^b^
2(5H)-Furanone **(11)**	0.159 ± 0.036 ^a^	0.108 ± 0.008 ^b^	0.094 ± 0.008 ^b,c^	0.058 ± 0.004 ^b,c^	0.048 ± 0.022 ^c^
*Trans*-whiskey lactone **(12)**	0.115 ± 0.025	n.d.	0.101 ± 0.013	n.d.	n.d.
2-phenylethanol **(13)**	n.d.	0.052 ± 0.003^a^	n.d.	0.087 ± 0.012^b^	n.d.
*Cis*-whiskey lactone **(14)**	0.707 ± 0.142 ^a^	n.d.	0.251 ± 0.041 ^b^	n.d.	n.d.
Cyclopropylcarbinol **(15)**	n.d.	n.d.	n.d.	n.d.	0.192 ± 0.196
4-hydroxy-2-methylacetophenone **(16)**	0.194 ± 0.049	0.091 ± 0.014	0.126 ± 0.015	0.121 ± 0.030	0.125 ± 0.078
Pyranone **(17)**	n.d.	n.d.	n.d.	n.d.	0.212 ± 0.217
Glycerin **(18)**	n.d.	n.d.	n.d.	3.554 ± 0.587	n.d.
Levulinic acid **(19)**	n.d.	n.d.	n.d.	n.d.	0.137 ± 0.151
p-acetylacetophenone **(20)**	n.d.	n.d.	n.d.	n.d.	0.268 ± 0.119
*Trans*-isoeugenol **(21)**	n.d.	n.d.	n.d.	0.289 ± 0.044 ^a^	0.199 ± 0.026 ^b^
Ethyl hydrogen succinate **(22)**	n.d.	n.d.	n.d.	0.211 ± 0.040	n.d.
2,3-dihydrobenzofuran **(23)**	n.d.	n.d.	n.d.	0.137 ± 0.021	0.175 ± 0.119
Benzoic acid **(24)**	n.d.	n.d.	n.d.	0.337 ± 0.008	0.471 ± 0.496
5-HMF **(25)**	1.206 ± 0.231	0.794 ± 0.040	0.834 ± 0.098	n.d.	1.347 ± 1.024
Methoxyeugenol **(26)**	n.d.	n.d.	n.d.	0.317 ± 0.043	0.845 ± 1.140
Vanillin **(27)**	2.491 ± 0.372 ^a^	1.990 ± 0.164 ^a,b^	1.815 ± 0.176 ^b^	0.483 ± 0.040 ^c^	0.138 ± 0.130 ^c^
Ethriol **(28)**	1.609 ± 0.326	0.511 ± 0.061	0.939 ± 0.082	n.d.	0.865 ± 0.956
Myristic acid **(29)**	n.d.	0.172 ± 0.058	0.122 ± 0.021	n.d.	0.700 ± 0.618
Palmitic acid **(30)**	1.095 ± 0.079	1.154 ± 0.029	0.768 ± 0.201	0.692 ± 0.040	1.759 ± 1.241
Syringaldehyde **(31)**	2.731 ± 0.390 ^a^	2.235 ± 0.205 ^a,b^	2.057 ± 0.130 ^b^	n.d.	0.453 ± 0.270 ^c^

Data are mean value ± standard deviation; values in the same row with different letters are significantly different (*p* < 0.05); n.d.: Not detected.

**Table 8:** Relative area values of volatile compounds determined by Stir-bar Sorptive Extraction–Gas Chromatography–Mass Spectrometry (SBSE-GC-MS) of wood extracts.

Compound	Extractant	American Oak	Spanish Oak	French Oak	Chestnut	Cherry
Ethyl butyrate	n.d.	d.	d.	0.010 ± 0.007	0.010 ± 0.002	0.020 ± 0.014
Isoamyl acetate	n.d.	d.	n.d.	0.265 ± 0.243	0.392 ± 0.170	0.258 ± 0.058
Limonene	n.d.	d.	0.031 ± 0.002	d.	d.	d.
Ethyl caprylate	0.693 ^a^	0.181 ± 0.024 ^b^	0.159 ± 0.003 ^b^	0.294 ± 0.157 ^b^	0.737 ± 0.253 ^b^	0.810 ± 0.359 ^b^
Ethyl caprate	1.523 ^a^	0.422 ± 0.039 ^b^^,c,d^	0.326 ± 0.017 ^b^^,c^	0.574 ± 0.414 ^b^^,d^	1.027 ± 0.640 ^e^^,f^	0.721 ± 0.336 ^e^^,f^
Isopentyl octanoate	n.d.	n.d.	n.d.	n.d.	n.d.	d.
Ethyl 2-phenyl acetate	n.d.	n.d.	n.d.	d.	0.057 ± 0.041	n.d.
Ethyl laureate	0.140	0.292 ± 0.029	0.228 ± 0.025	0.422 ± 0.342	0.395 ± 0.267	0.295 ± 0.212
Caprylic acid	0.191	0.163 ± 0.089	0.088 ± 0.018 ^a^	0.147 ± 0.062	0.184 ± 0.059 ^b^	0.202 ± 0.068 ^b^
Ethyl myristate	0.078	0.095 ± 0.036	d.	d.	0.266 ± 0.255	0.202 ± 0.181
Capric acid	0.214	0.215 ± 0.163	d.	d.	0.090 ± 0.040	0.091 ± 0.032
Ethyl palmitate	0.233	0.261 ± 0.207	d.	d.	0.152 ± 0.077	0.119 ± 0.052
Ethyl 9-hexadecenoate	0.035	d.	d.	d.	d.	d.
Ethyl stearate	n.d.	d.	n.d.	n.d.	n.d.	n.d.
Lauric acid	0.731 ^a^	0.131 ± 0.051 ^b^	0.054 ± 0.038 ^c^	0.090 ± 0.028 ^b,c^	0.085 ± 0.014 ^b,c^	0.040 ± 0.028 ^c^
Myristic acid	0.057 ^a^	0.263 ± 0.085 ^b^	0.180 ± 0.016 ^a,b,c^	0.200 ± 0.081 ^b,c^	0.170 ± 0.051 ^a,c^	0.138 ± 0.023 ^a,c^
Pentadecanoic acid	n.d.	d.	d.	0.097 ± 0.053	0.069 ± 0.019	0.079 ± 0.014

Data are mean value ± standard deviation; values in the same row with different letters are significantly different (*p* < 0.05); n.d.: Not detected; d.: Detected.

**Table 9:** Phenolic compounds contents (mg L^−1^) and total polyphenol index (TPI) (mg gallic acid equivalent (GAE) L^−1^) of wood extracts.

Compound	American Oak	Spanish oak	French oak	Chestnut	Cherry
Gallic acid **(1)**	0.98 ± 0.14 ^a^	3.35 ± 0.17 ^b^	1.69 ± 0.15 ^c^	0.23 ± 0.06 ^d^	0.17 ± 0.32 ^e^
Hydroxymethylfurfural **(2)**	0.28 ± 0.06 ^a^	0.44 ± 0.09 ^b^	0.40 ± 0.05 ^b^	0.08 ± 0.04 ^c^	n.d.
Furfural **(3)**	0.10 ± 0.01 ^a^	0.41 ± 0.03 ^b^	0.33 ± 0.04 ^c^	n.d.	n.d.
Caffeic acid **(4)**	n.d.	n.d.	n.d.	0.28 ± 0.02	n.d.
Vanillic acid **(5)**	1.10 ± 0.19 ^a^	0.59 ± 0.05 ^b^	1.48 ± 0.019 ^c^	0.55 ± 0.11 ^b^	n.d.
Syringic acid **(6)**	0.32 ± 0.09 ^a^	0.46 ± 0.04 ^b^	0.36 ± 0.05 ^a^	0.14 ± 0.01 ^c^	0.08 ± 0.16 ^d^
Vanillin **(7)**	0.87 ± 0.04 ^a^	2.00 ± 0.08 ^b^	1.46 ± 0.04 ^c^	0.39 ± 0.03 ^d^	n.d.
Syringaldehyde **(8)**	0.90 ± 0.03 ^a^	2.18 ± 0.07 ^b^	1.37 ± 0.07 ^c^	0.25 ± 0.05 ^d^	n.d.
Ellagic acid **(9)**	4.65 ± 0.10 ^a^	8.82 ± 0.59 ^b^	8.03 ± 0.50 ^c^	3.36 ± 0.15 ^d^	n.d.
Coniferylaldehyde **(10)**	1.21 ± 0.04 ^a^	2.51 ± 0.07 ^b^	1.83 ± 0.06 ^c^	0.19 ± 0.05 ^d^	n.d.
Sinapaldehyde **(11)**	1.53 ± 0.02 ^a^	4.59 ± 0.08 ^b^	2.93 ± 0.10 ^c^	0.25 ± 0.05 ^d^	n.d.
Total Phenolic Index	184.61 ± 1.68 ^a^	355.26 ± 3.51 ^b^	285.15 ± 10.52 ^c^	120.28 ± 1.20 ^d^	252.34 ± 0.72 ^e^

Data are mean value ± standard deviation; values in the same row with different letters are significantly different (*p* < 0.05); n.d.: Not detected.
